# A comparative study of the drying evolution and dried morphology of two globular proteins in de-ionized water solutions†

**DOI:** 10.1039/d0ra01748e

**Published:** 2020-04-30

**Authors:** Anusuya Pal, Amalesh Gope, Ari S. Athair, Germano S. Iannacchione

**Affiliations:** Department of Physics, Order-Disorder Phenomena Laboratory, Worcester Polytechnic Institute Worcester MA 01609 USA apal@wpi.edu; Department of English, Tezpur University Tezpur Assam 784028 India

## Abstract

Pattern formation in drying protein droplets continues to attract considerable research attention because it can be linked to specific protein–protein interactions. An extensive study of the drying evolution and the final crack patterns is presented, highlighting the concentration dependence (from 1 to 13 wt%) of two globular proteins, lysozyme (Lys) and bovine serum albumin (BSA), in de-ionized water. The drying evolution starts with a constant contact radius mode and shifts to a mixed mode where both fluid front and contact angle changes. The contact angle monotonically decreases, whereas, the fluid front exhibits two regimes: an initial linear regime and a later non-linear regime. Unlike the linear regime, the non-linear regime is faster for Lys droplets. This results in the formation of a “mound”-like structure in the central region. A new feature, a “dimple” is observed in this mound which is found to be dependent on the initial concentration. The different crack morphology of BSA and Lys depends strongly on the initial state of the solution and can be interpreted using a simple mechanical model. In fact, when dried under uniform conditions (surface, humidity, temperature, droplet diameter, *etc.*), the evolution and the final pattern displays as a fingerprint of the initial state.

## Abstract 2

Signature pattern formation in drying globular protein solution droplets: understanding self-assembled macroscopic states as indicators of the initial microscopic states.

## Graphical abstract

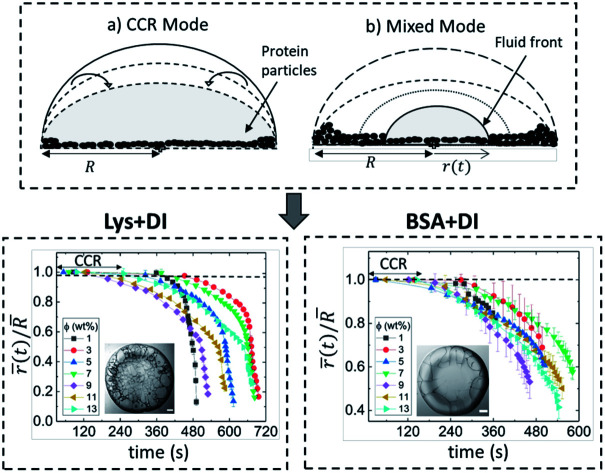

## 1 Introduction

A colloidal droplet deposited on a surface either spreads over the surface or remains as it is depending on the wettability of the surface. Whatever the case, the droplet endures a whole range of interfacial phenomena (wetting dynamics, adsorption, and adhesion), internal flow (diffusion and convection), and particle–substrate interactions during the solvent evaporation (drying).^1,2^ The pattern formation of a bio-colloidal droplet, even on an ideal surface, is exposed to additional complexity during the drying process. This complexity arises primarily due to strong and potentially competing interparticle interactions that govern the particle aggregation and self-assembly. Furthermore, some bio-colloidal droplets such as blood and plasma serum are ubiquitous, and, most notably, are used in medical diagnostics and forensics analysis.^3–6^ Studies on drying droplets reveal that the evolution and the emerging patterns depend on multiple factors including the nature of the solute particles (size, chemical composition initial concentration), different type of substrates (hydrophilic, hydrophobic), geometry, substrate wetting, and various drying conditions (temperature, pH, relative humidity).^7–11^ Accordingly, it turns out to be essential to understand the effects of these factors on the drying and dried bio-colloidal droplets in order to compare (and explain) the macroscopically observed behavior with the initial microscopic state of the constituent particles.

Owing to its significance in many potential applications, the drying evolution and the morphological patterns of dried plasma and blood have been examined by several researchers.^3,4,6^ Essential mechanisms of spreading, gelation, and crack formation of these droplets are presented in recent review articles by Brutin *et al.*^12^ and Chen *et al.*^13^ An aqueous solution of protein drying droplets may similarly be substantiated as a prolific system of biological relevance. The aqueous solution of proteins is far less complicated than blood and plasma. In a recent study, the drying droplets of raw egg-white protein solutions have been explored which validate the dependence of daisy and wavy-ring crack patterns on the initial protein concentrations.^14^

It is worth mentioning that commercially available high-quality globular proteins such as bovine serum albumin (BSA) and lysozyme (Lys) have also attracted the attention of many researchers. Many studies investigated the temporal drying process and their resulting patterns. Despite the intense research, most of the work on protein–solvent systems is primarily confined to either a dilute regime of protein concentration and/or the involvement of the salts into the systems.^15–19^ The dilute regime of the initial protein concentration is well explored in the drying droplets; however, not beyond the range of 60 mg mL^−1^. Researchers also attempted to explore the drying evolution and the crack distribution of these proteins dissolved in (de-ionized) water under ambient conditions.^15,20^ Gorr *et al.*^15^ studied the time evolution and the morphological patterns of Lys dissolved in water, varying the initial concentration from 0.1 (1 mg mL^−1^) to 1 wt% (10 mg mL^−1^). This study concluded that all the drops exhibit a “coffee-ring” effect.^15,21^ The volume fraction of the Lys is found to be linearly dependent on its initial concentration; however, the morphology does not show any significant changes in the ring’s height and width. They also reported a “mound”-like feature in the central region and observed a few surface cracks in the given concentration range. Carreón *et al.*^20^ investigated the mixture of BSA and Lys dissolved in water and mainly focused on their interactions. They also conducted experiments with denatured BSA and Lys proteins and their mixtures at different relative concentrations. The folding and unfolding of these proteins and their structural (morphological) alternation are also discussed in their paper. They concluded that the formation of the crystal clusters and dendrite structures are independent of the external salts.

In this paper, two fundamental questions about the drying evolution and the resulting patterns of the dried droplets of aqueous solutions of commercially available globular proteins, are addressed. This article aims to explore (i) the role of the protein properties (in terms of mass, composition, configuration, and size); and examine, (ii) the effect of higher initial protein concentration (above 60 mg mL^−1^ and up to 150 mg mL^−1^) on the aggregation process. The inclusion of the higher-initial protein concentration is essential since the higher concentrations enable us to explore how the excessive aggregation of these proteins play a role in relieving mechanical stress during the drying process, and the crack formation patterns. It is worth mentioning that no studies to date have attempted to investigate these fundamental queries at the concentration ranges we considered in this paper. To the best of our knowledge, we did not come across any experimental evidence that compares the drying evolution of the protein droplets in terms of the contact angle and the fluid front. To address this gap, two proteins, BSA and Lys, are chosen in this study. The droplets are prepared using de-ionized water (DI) that avoids ion-mediated effects and exposes the protein–protein interactions. Furthermore, the inclusion of DI under ambient conditions ensures that the functionality of the proteins is not affected by any external factors such as pH, temperature, *etc.*

The drying evolution and the emerging patterns are then examined at the initial protein concentration ranging from 1 (10 mg mL^−1^) to 13 wt% (150 mg mL^−1^). Each solution sample is deposited as a ∼2 mm diameter droplet on a glass slide to record the temporal variation of contact angle, fluid front, and to capture the image of the final dried state. An image processing technique is employed to extract the distribution of the distance between consecutive cracks (crack spacing) in the protein droplets. To ensure the reliability of the visual observations, an appropriate statistical test is used on the obtained crack-spacing data. A physical mechanism is proposed in this paper to relate the dried morphology with the nature of the initial protein solutions. Finally, the crack patterns are interpreted in terms of a simple mechanical stress model to explain the presence of a crack hierarchy in the Lys (not seen in BSA) droplets.

The paper is structured as follows. Following this introduction, section 2 describes the materials and the experimental methodology adopted in this paper. Section 3 presents the process of the drying evolution, the morphology, the physical and mechanical mechanism, and the statistical findings on the dried crack patterns in both protein droplets. The results and the significant findings of this paper are discussed in this section. Finally, the results are concluded in section 4.

## 2 Materials and experimental methodology

BSA and Lys are well-studied water-soluble globular proteins. BSA is a representative blood protein primarily derived from cows. It is four times heavier than Lys. BSA has a molecular mass of ∼66.5 kDa with an ellipsoid shape of dimensions 4.0 × 4.0 × 14.0 nm^3^.^22^ Lys, on the other hand, can be observed in human mucosal secretions such as saliva, tears, *etc.* It consists of a molecular mass of ∼14.3 kDa, and a roughly ellipsoid shape of dimensions 3.0 × 3.0 × 4.5 nm^3^.^23^ Lys is made up of 129 amino acids whereas, this blood protein contains 581 amino acids in a single polypeptide chain. The isoelectric point of BSA is 4.7 which enables it to carry a net negative charge under the present conditions of the study (pH of ∼7). The isoelectric point of 11.1 in Lys, on the other hand, allows it to carry a net positive charge. The globular shape and stability of these proteins are attributed to the disulfide bridges (17 in BSA and 4 in Lys), hydrogen bonds, and hydrophobic interactions among the amino acids.^24^ It is to be noted that the globular nature of the overall tertiary structure of these proteins is maintained in this present study. The synthetic polymer-based colloidal behavior of these proteins is only possible when these are completely denatured (or their structures are relaxed). Therefore, this study is much more complicated and difficult to interpret than other polymer-based droplet studies.

The commercial lyophilized BSA and the Lys are obtained from Sigma Aldrich, USA (Catalog no. A2153 and L6876 respectively). The protein samples are used without any further purification. ∼150 mg of each BSA and Lys were measured and separately dissolved in 1 mL of de-ionized water (Millipore, resistivity of 18.2 MΩ cm) to create the protein stock solutions, BSA + DI and Lys + DI at a concentration of 13 wt%. Each stock solution was diluted to prepare concentrations of 1, 3, 5, 7, 9, and 11 wt%. To make samples combining protein and liquid crystal (LC), 4-cyano-4′-pentyl-biphenyl was purchased from Sigma Aldrich, USA (Catalog no. 328510). The LC was heated at ∼39 °C, and a volume of ∼10 μL was added to the prepared protein solutions of 9 wt%. The sample sets were used in the next few hours of their preparation time. We used the factory-fresh microscopic glass slides as the substrates, which have minimal exposure to the environment prior to the actual experiments. These slides were rinsed with ethanol and dried. Thus, every droplet precisely contained the same substrate conditions and a uniform reproducibility in terms of the circularity of the droplets and their pinning effects was observed. A volume of 1 ± 0.2 μL of the sample was pipetted to form a circular droplet of ∼2 mm diameter. For strip-like geometry, two carbon tapes were placed in parallel on the glass substrate by keeping a separating distance of ∼2 mm, and a volume of ∼5 μL of the protein samples was pipetted. The samples were left to dry under a bright-field microscope (Leitz Wetzlar, Germany) with a 5× objective lens in ambient conditions (room temperature of ∼25 °C and relative humidity of ∼50%). The total time of the drying process for all droplets was roughly around 10–20 minutes.

The captured images were analyzed using ImageJ^25^ software. The time-lapsed images of the drying process were obtained every two seconds. The start time of the clock was the time of the deposition point of the droplet on the glass slide. The fluid front radius (*r*(*t*)) was measured by tracking the distance from the center to the edge of the front under the microscopy during the drying evolution. These radii were measured repeatedly ten times at each time (*t*), and the averaged values (*r̄*(*t*)) were computed. The spiral crack study was conducted using a 50× objective lens. The images of dried droplets were captured over 24 hours using side-illuminated bright-field microscopy (since a few cracks appeared after the visible drying process^26^). The *stitching plugin*^27^ of ImageJ was used to redraw the complete image of the dried droplets. The stitched bright-field images were converted into high contrast images. The circular cut lines were drawn using the *oval profile* plugin of ImageJ. The intensity values (255 for pixels depicting crack lines and 0 for elsewhere) were plotted as a function of arc-length. A script with “Array and Maxima” was used to determine the positions of the maximum intensity values to estimate the spacing between the consecutive cracks (*x*_c_). The detailed processing can be found in our previous paper.^28^ The same process was repeated for two other droplets deposited from the same sample set at each concentration (*ϕ*) to ensure the reproducibility of the final morphology. The data were aggregated to yield an overall crack spacing *x*_c_ at each *ϕ*. It is to be noted that this crack spacing analysis is conducted only at the peripheral region where the cracks of both protein droplets are present. Furthermore, the contact angle goniometer (Model 90, Ramé-hart Instrument Co. USA) was used to monitor the contact angle (*θ*(*t*)) measurements of the prepared protein samples during the drying process.

## 3 Results and discussion

### 3.1 Time evolution of drying droplets

#### 3.1.1 Bovine serum albumin: BSA + DI

**Fig. 1:**
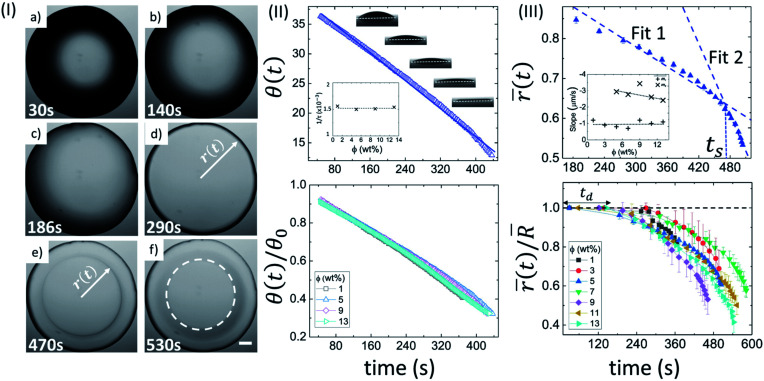
Drying evolution of a BSA droplet: (I) top-view of the droplet through optical microscopy at *ϕ* of 5 wt% obtained during different time intervals (30 s, 140 s, 186 s, 290 s, 470 s and 530 s). The white dashed circle in (f) exhibits the “coffee-ring” formation. The scale bar represents a length of 0.20 mm. (II) The top panel shows the variation of contact angle (*θ*(*t*) in degrees) at *ϕ* of 5 wt%. The solid line specifies the fitted function. The inset shows the variation of the characteristic fitting parameter (1/*τ* in s^−1^) at different *ϕ*. The bottom panel displays the temporal variation of the normalized contact angle (*θ*(*t*)/*θ*_0_)). (III) The top panel reveals the variation of mean fluid front radius (*r̄*(*t*) in mm) at *ϕ* of 5 wt%. The error bars represent the standard deviation. A representative fit of two linear models is made; *t*_s_ signifies the time point where both the linear fits merge. The inset shows the dependence of the slope values of linear fit 1 (*m*_1_) and 2 (*m*_2_). The bottom panel indicates the temporal variation of normalized mean fluid front radius (*r̄*(*t*)/*R̄*) at different *ϕ*. *t*_d_ is the “dead” time up to which the fluid front radius (*r*(*t*)) is equal to the radius of the droplet (*R*).

Fig. 1(I) shows the top view of the drying evolution of a BSA droplet at the initial concentration (*ϕ*) of 5 wt% captured through optical microscopy. As soon as the first image of the deposited droplet is captured, a symmetrical dark (black) shade is observed near the periphery of the droplet. With the progression of time, the dark shade changes to the bright (gray) shade (Fig. 1(I)a–c). The periphery of the droplet is found to be pinned to the glass substrate throughout the drying process. After ∼3 minutes, the fluid front starts receding from the periphery to the center of the droplet. The radius (*r*) of this front is measured with the progression of time, (*t*) (Fig. 1(I)d and e). The movement allows the particles to be deposited along each receding line and eventually forms a peripheral ring (shown by a white dashed circle in Fig. 1(I)f). It is to be noted that a few cracks in the BSA droplets up to *ϕ* of 5 wt% are formed within 24 hours, which are shown in the following section.

Fig. 1(II) depicts the side view of the drying evolution of a BSA droplet at different *ϕ* observed with the contact angle goniometer. The contact angle (*θ*) and the height at the center of the droplet (*h*) are found to be 37 ± 2° and 0.35 ± 0.20 mm, respectively, within ∼50 seconds of the deposition of the droplet. The radius (*R*) of the droplets is 1.1 ± 0.2 mm, and therefore, the gravitational effects can be negligible. Furthermore, the macroscopic shape of the droplet can be approximated as the spherical-cap geometry, which is based on the assumption that *h* ≪ *R*. The black shade, observed at *ϕ* of 5 wt% in Fig. 1(I) is due to the spherical-cap shape of the droplet. The change from the black to the gray shade occurs when the contact angle (*θ*) reaches a threshold value. The top panel of Fig. 1(II) shows the variation of *θ* at *ϕ* of 5 wt%, and *θ*(*t*) is found to consistently reduce with time. This suggests that the drying process occurs under a continuous evaporation limit. To validate this limit, *θ*(*t*) is fitted to a linear function: *θ*(*t*) = *θ*_0_(1 − *t*/*τ*), where, *θ*_0_ is the initial *θ* at *t* = 0, and 1/*τ* is a characteristic rate. For *ϕ* of 5 wt%, *θ*_0_ and 1/*τ* are found to be 39.96 ± 0.08° and 0.001500 ± 0.000005 s^−1^, respectively, with *R*^2^ = 0.995. The 1/*τ* is observed to be independent of *ϕ* (inset of Fig. 1(II)). A complete description of all the fit parameters is tabulated in Table T1 of the ESI.† The normalized contact angle is calculated by dividing *θ*(*t*) with the *θ*_0_ (obtained from the fitting equation). The individual normalized *θ* decay curves at different *ϕ* are found to collapse to a master curve when the data is plotted. This is shown in the bottom panel of Fig. 1(II).

In this context, it is interesting to compute the fluid front radius and its dependence on *ϕ*. Fig. 1(III) shows the evolution of the mean fluid front radius (*r̄*(*t*)) in a BSA droplet at different *ϕ*. The top panel shows the variation of *r̄*(*t*) at *ϕ* of 5 wt% exhibiting two distinct regimes: a slow, initial linear regime, and a subsequent non-linear, fast regime. Two linear fits are made on the respective linear and non-linear regimes, and a characteristic time, *t*_s_ (the time point at which two linear fits intersect) is introduced. It is to be noted that the linearity of *r̄*(*t*) deviates after the peripheral ring formation (Fig. 1(I)e and top panel of Fig. 1(III)). The inset of Fig. 1(III) compares the slope values (*m*_1_ and *m*_2_) obtained from the linear fits in the respective regimes at each *ϕ*. The negative sign in the slope values confirms the reduction of the mean radius (*r̄*(*t*)) with time. For 1 and 3 wt%, the linear fit in the non-linear regime cannot be achieved due to a swift and non-uniform movement, resulting in a lower number of data points to quantify. On average, the velocity of the fluid front in the linear and non-linear regime is found to be 0.99 ± 0.20 μm s^−1^ and 2.83 ± 0.38 μm s^−1^ respectively. *m*_2_ decreases from ∼3 to ∼2 μm s^−1^ with the increase of *ϕ*. The bottom panel of Fig. 1(III) displays the normalized radius (obtained by dividing the *r̄*(*t*) with the mean radius of the droplet, *R̄*). In the early stage of the drying evolution, *i.e.*, up to ∼240 seconds, the radius remains constant, (*r̄*(*t*)/*R̄* = 1) for all the *ϕ*. This time is labeled as the “dead” time (*t*_d_) where only the contact angle changes without disturbing the radius. A complete description of all the measured and fit parameters is tabulated in Table T2 of the ESI.†

#### 3.1.2 Lysozyme: Lys + DI

**Fig. 2:**
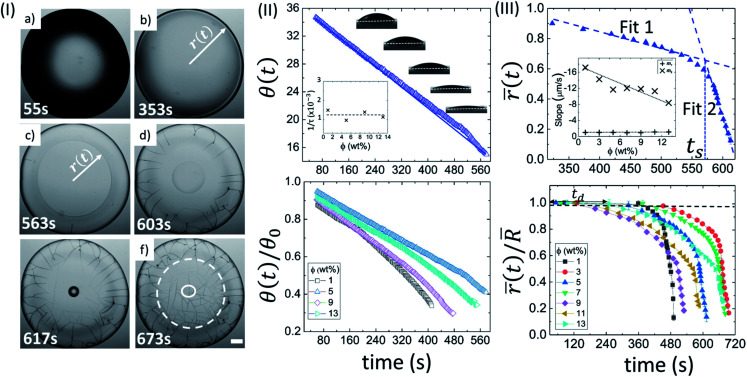
Drying evolution of a Lys droplet: (I) top-view of the droplet through optical microscopy at *ϕ* of 5 wt% during different drying intervals (55 s, 353 s, 563 s, 603 s, 617 s and 673 s). The white dashed circle in (f) shows the “coffee-ring” formation. The solid circle exhibits the “mound”-like structure. The scale bar represents a length of 0.20 mm. (II) The top panel confirms a variation of contact angle (*θ*(*t*) in degrees) at *ϕ* of 5 wt%. The solid line shows the fitted function. The inset shows the variation of the characteristic fitting parameter (1/*τ* in s^−1^) at different *ϕ*. The bottom panel displays the temporal variation of the normalized contact angle (*θ*(*t*)/*θ*_0_)). (III) The top panel reveals the variation of the mean fluid front radius (*r̄*(*t*) in mm) at *ϕ* of 5 wt%. The error bars represent the standard deviation. A representative fit of two linear models is made; *t*_s_ signifies the time point where both the linear fits merge. The inset represents the dependence of the slope values of linear fit 1 (*m*_1_) and 2 (*m*_2_). The bottom panel indicates the temporal variation of the normalized mean fluid front radius (*r̄*(*t*)/*R̄*) at different *ϕ*. *t*_d_ is the “dead” time up to which the fluid front radius (*r*(*t*)) is equal to the radius of the droplet (*R*).

Akin to BSA, a Lys droplet at *ϕ* of 5 wt% also shows a dark shade near the periphery of the droplet (Fig. 2(I)a). The dark shade diminishes, as the fluid front starts receding from the periphery after ∼6 minutes, and forms a ring (Fig. 2(I)b–d). Interestingly, a sharp spot around the center appears and forms a “mound”-like structure. The water starts drying from that mound, and finally, a “dimple” appears in the existing structure. Simultaneously, the radial cracks grow near the periphery and come into contact with each other through the orthoradial cracks (Fig. 2(I)d–f). The white dashed circle displays the peripheral ring and the solid circle depicts the mound and the dimple structures (Fig. 2(I)f). Unlike BSA, most of the cracks appeared during the visible drying process at *ϕ* of 5 wt%.

Fig. 2(II) shows the side view of the drying evolution of a Lys droplet at different *ϕ*. It is to note that different Lys concentrations might affect the surface tension of the solutions. However, our first measured value of *θ* during the contact angle measurements at all *ϕ* is found to be 37.0 ± 1.6°. This measurement tempted us to conclude that the effect is not significant enough for the unique pattern formation. The *θ* at *ϕ* of 5 wt% during the drying process reduces consistently (top panel of Fig. 2(II)). *θ*_0_ and 1/*τ* are found to be 36.66 ± 0.01° and 0.0009740 ± 0.0000007 s^−1^, respectively, with *R*^2^ = 0.999. Similar to BSA, the characteristic rate (1/*τ*) is found to be independent of *ϕ*. A complete description of all fit parameters is tabulated in Table T3 of the ESI.† Unlike BSA, the normalized contact angle data shows that the individual decay curves at different *ϕ* start deviating from each other towards the very end of the process (bottom panel of Fig. 2(II)).

The top panel of Fig. 2(III) displays the evolution of the (*r̄*(*t*) at *ϕ* of 5 wt% in Lys droplet. And, the bottom panel depicts the evolution of the normalized mean fluid front radius (*r̄*(*t*)/*R̄*) at different *ϕ*. It is noted that this movement in BSA droplets could be tracked only up to the point where the radius just passes through the peripheral ring. Unlike BSA, this movement in the Lys droplets could be tracked till the “mound”-like structure around the central region of the droplet formed. This causes the range of the *r̄*(*t*)/*R̄* data to be 1 to ∼0.1. The presence of a linear and a subsequent non-linear regime is commonly observed in the fluid front movement at every *ϕ* in both the Lys and BSA droplets. On average, the velocity of the fluid front in the linear and non-linear regime of these Lys droplets is found to be 1.00 ± 0.08 µm s^−1^ and 12.36 ± 2.73 µm s^−1^, respectively. A sharp dependence of the slope values in regime 2 with *ϕ* is observed, *m*_2_ decreases from ∼17 to ∼8 µm s^−1^. A complete description of all the measured and fit parameters is tabulated in Table T4 of the ESI.†

#### 3.1.3 A physical mechanism

**Fig. 3:**
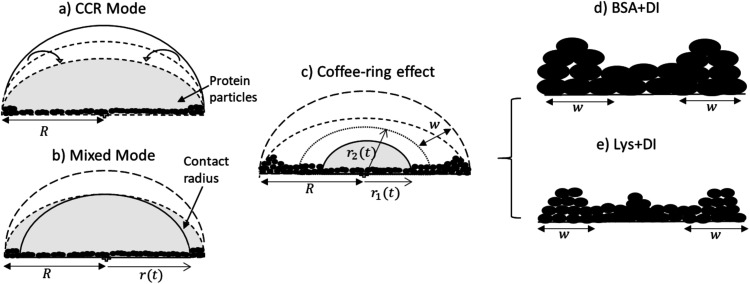
Side view of the drying evolution: (a) CCR mode is observed at an early stage of drying where only the contact angle decreases. The fluid front radius (*r*) remains unchanged during this time and is equal to the radius (*R*) of the droplet. The protein particles interact with the substrate during this stage. (b) In this mixed mode stage, along with the contact angle, the fluid front starts receding from the periphery of the droplet. (c) The fluid front moves, deposits more particles, and forms a ring of width *w*. It moves further when the radius of the fluid front just passes the ring width to the new position (*r*_2_). *r*_1_ indicates the movement towards the central region of the droplet, leading to a complimentary morphology. (d) BSA + DI, without any “mound”-like structure and (e) Lys + DI, the presence of a “mound” could be observed in the central region.

The underlying physical mechanism of the drying evolution and the visible difference in terms of the morphology of the droplets is demonstrated in Fig. 3. The deposited droplet goes through a convective flow where the constituent particles tend to interact (adsorb) with the substrate during the early drying stage. The evaporation rate is observed to be the highest at the three-phase contact line (solid–vapor–liquid) due to the curvature of the circular droplet. This process drives the flow to compensate the higher rate of mass loss near the periphery compared to the central part of the droplet. This early stage depicted in Fig. 3a shows the constant contact radius mode (CCR) in which no fluid front movement is observed; however, the height and the contact angle get considerably reduced. During this time-frame, the protein particles form an inhomogeneous film and (subsequently) a fluid front starts moving on this film from the periphery to the center which marks the beginning of the next stage. The mixed mode *i.e.*, the movement of both the contact angle and the fluid front is found at this stage. The front seems to deposit more protein particles as it moves (Fig. 3b). A bulge (popularly known as “coffee ring” effect^21^) at the periphery of the droplet is noticed during this fluid front movement. The bulge is believed to form due to the excess deposition of the particles which could be seen in Fig. 3c. The drying process up to this point, *i.e.* the formation of this peripheral ring, is observed to be similar for both protein droplets (Fig. 1 and 2). However, the formation of a “mound”-like structure (Fig. 3d and e) in the Lys droplets (a similar phenomenon is observed in ref. 15 and 20) creates a visible difference in the drying evolution of both protein droplets. The comparison of the drying rates of these different-sized protein particles may reveal new insights. To map the rate of water loss with the change in morphology during the drying process, it is vital to explore the possible reasons behind the similarities and dissimilarities for both protein droplets in terms of the parameters (*t*_d_, *t*_s_, *m*_1_, and *m*_2_) extracted from the fluid front movement.

*t*_d_ indicates the (early stage) time point of the drying process where the protein particles in both droplets experience convective flow. During this flow, these particles first tend to interact (adsorb) with the substrate. The usage of de-ionized water in the present study enables us to avoid ion-mediated effects and does not influence the conformational states (or the functionality) of these proteins. This means that the globular nature of the overall protein structure during the drying process is maintained. During adsorption of these proteins on the glass substrate, one can expect different BSA-glass and Lys-glass interactions. This expectation can be due to the fact that the BSA and the Lys proteins carry an opposite net charge whereas, the glass (substrate) is negatively charged. However, we also need to consider that the hydrophobic residues are buried inside the protein core, and numerous positively and negatively charged residues in a protein’s surface are exposed. Though the overall charge of BSA (or Lys) is negative (or positive), means that the BSA (or Lys) will prefer to adsorb on the negatively charged glass substrate with its positively charged residues. Therefore, the overall interaction of BSA-glass or Lys-glass might not be altered, while there is a high probability of having different BSA–BSA or Lys–Lys interactions. This is because these charged residues help orient these protein particles in such a way that one particle gets influenced by the neighboring particle. With time, the protein–protein interactions start to become dominant over the protein–substrate interactions, and assist in forming the protein film on the substrate, and finally could be responsible for determining these unique patterns. So, *t*_d_ is the time in which protein particles interact with the substrate and interact with other proteins to form a film. The constant rate of the evaporation validates the similar trend in this CCR mode up to the time point *t*_d_.

As time passes during the drying process, we observed a fluid front to recede from the periphery to the center of the droplet. We quantified the velocity of the front movement. The average speed of first linear fit (*m*_1_) is found to be 0.99 ± 0.14 μm s^−1^, which is independent of the initial concentration and the type of protein. Considering the trends observed in *m*_1_, it could be concluded that similar mass transfer mechanisms have emerged in the linear regime. This assumption makes sense because there is enough water on the front surface at this stage, and the front behaves as if it is a water-pool and hardly feels the presence of any protein particles. Subsequently, we have observed a transition from the linear to the non-linear regime in the front movement (Fig. 1(III) and 2(III)). A linear fit on the linear and non-linear regimes was made. *t*_s_ signifies the time point where both these linear fits merge. Interestingly, it can be physically interpreted as the time when the fluid front moves from *r*_2_ to *r*_1_ (Fig. 3c), *i.e.*, the time point of the movement from the edge of the peripheral ring towards the central region of the droplet. The *t*_s_ for all *ϕ* is found to be within ±20 s from this ring formation.

Once the fluid front passes this peripheral ring, the fluid no longer resembles a water-pool. The continuation of the water evaporation process leads to the presence of more protein particles than water. In this context, we observed that the velocity of the second linear fit (*m*_2_) is dependent on the initial concentration and this is different for both protein droplets. The differences observed in *m*_2_ are probably due to different self-assembling interactions, which is dependent on the unique physical characteristics of these proteins. Given the globular nature of these proteins, we know that these proteins are different in terms of their net charged states, molecular shape, weight and disulfide bridges. Since the pH of the system is unchanged, it is beyond the scope of this paper to conclude the mobility effects that emerged due to the individual charged residues present in the protein during the fluid front movement. However, this mobility can easily be interpreted in terms of their weight, shape and bridges. BSA particles are mostly restricted to flow with the fluid front due to high molecular weight (∼66.5 kDa) and a high aspect ratio (major/minor axis = 3.5). Moreover, the presence of 17 disulfide bridges in BSA provides a compact network between BSA–BSA particles; it will prefer to be deposited within the existing film-layer in the droplet. This results in a few left-over BSA particles to be carried with the water during the later stage of the fluid front movement. In contrast, Lys could be thought of as a squishy sphere (aspect ratio = 1.5) with low molecular weight (∼14.3 kDa). The presence of the lower disulfide bridges (17 for BSA and 4 for Lys) results in a weak network among these Lys particles, and it triggers the Lys particles to be carried away with the fluid front. The water content of the fluid decreases with the progression of time, and a large number of Lys particles are left behind. These (left-out) particles eventually accumulate around the center and forms the “mound”-like structure. We believe that some water is trapped in the mound. Therefore, a dimple is noticed when the Lys particles fall out of the solution as this entrapped water evaporates.

The movement of the fluid front appears to slow down with the increase of *ϕ* (number of particles), even though the movement continues to carry and deposit the Lys particles at each line of the fluid front. The concentration dependence of the mound and the peripheral ring on different protein types will be discussed later. At the final stage of the drying process, the water-loss in the droplet induces high mechanical stress leading to the formation of different crack patterns, which will be discussed in the next subsection. The movies of the drying process in both protein droplets are available in the ESI,† V1 for BSA + DI and V2 for Lys + DI; both videos are recorded at *ϕ* of 5 wt%.

### 3.2 Morphology of dried droplets

**Fig. 4:**
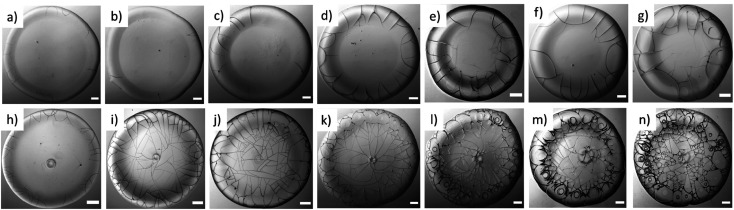
Gray images of the protein dried droplets: BSA at *ϕ* of (a) 1, (b) 3, (c) 5, (d) 7, (e) 9, (f) 11, and (g) 13 wt%; Lys at *ϕ* of (h) 1, (i) 3, (j) 5, (k) 7, (l) 9, (m) 11, and (n) 13 wt%. The images of the BSA droplets reveal the presence of the cracks near the periphery region, whereas, the images of the Lys droplets confirm that the cracks are spread and connected from *ϕ* of 3 wt%. The “dimple” and the “mound”-structure can be observed in the Lys droplets at every *ϕ*. The spirals are noticed in the Lys droplets from *ϕ* of 9 wt%. The scale bar represents a length of 0.20 mm.

Fig. 4a–g and h–n represent the morphology of the dried droplets in BSA and Lys, respectively, both the common and distinctive properties are identified. A few common characteristics of both dried droplets include: (1) the presence of a peripheral ring at a greater height than the central region. This greater height can be viewed from the one-sided dark shadowy shade due to side illumination; (2) the cracks are observed in both the droplets; however, the distribution and the nature of the cracks differ.

A few distinct morphological patterns are observed in the protein droplets: (1) in BSA droplets, mostly the radial cracks are observed, whereas different types of cracks such as radial, wavy, spiral, *etc.* make the Lys droplets a chaotic system. (2) There are almost no cracks in the central region of the BSA droplet, whereas the cracks are present throughout the Lys droplets. However, cracks are found only in the periphery of the Lys droplets at *ϕ* of 1 wt%. (3) The morphology of the central region has a mound and dimple structures in Lys, but no such structures in the BSA droplet. (4) The increase of cracks with the increase of *ϕ* is clearly observed in BSA. The cracks are mostly equally spaced and countable. (5) A thin hair-like structure is observed at the termination of each crack (from 7 to 13 wt%) in BSA droplets. In contrast, the cracks are well-connected and form a uniform domain in every Lys droplet (except the *ϕ* of 1 wt%). (6) The presence of (few) circular and (multiple) spiral cracks in Lys droplets from 9 to 13 wt% makes the morphology very different in the highly concentrated regime, from that in BSA droplets. The possible reasons are discussed in the mechanical interpretation section.

#### 3.2.1 Profilometry

**Fig. 5:**
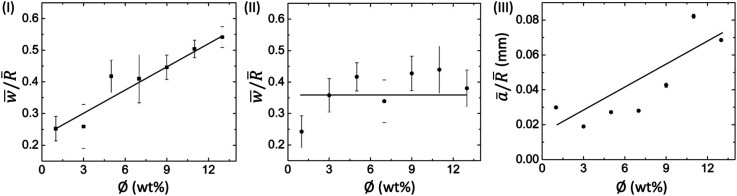
Profilometric measurements of protein droplets: variation of the mean peripheral ring width (*w̄*) normalized to the mean radius of the droplet (*R̄*) with *ϕ* for (I) BSA + DI, and (II) Lys + DI. (III) Variation of the mean area of the “mound”-like structure (*ā*) normalized to *R̄* with *ϕ* in the Lys droplet. The error bars correspond to the standard deviation obtained from multiple measurements.

Fig. 5(I) and (II) show the variation of a dimensionless quantity, the mean peripheral ring width (*w̄*) divided by the mean radius of the droplet (*R̄*) with *ϕ* for BSA and Lys droplets, respectively. It is observed that the ring width is directly proportional to *ϕ*, *i.e.*, with the increase of *ϕ*, this ring width is expanded further in these BSA droplets (Fig. 5(I)). The number of BSA particles increases with the evaporation of water. This process triggers additional deposition of the particles at the droplet periphery with the upsurge of *ϕ*. On the contrary, the Lys droplet is found to be constant and independent of the variation of *ϕ*. An almost equal quantity of Lys particles is deposited in the ring, and most of the free particles are carried towards the center (and form a mound structure). Fig. 5(III) shows the areal dependence of this mound structure. The mean area *ā* is normalized with *R̄*, and the *ā*/*R̄* at different *ϕ* in the Lys droplet is plotted. The linear dependence with *ϕ* makes it evident that most of the Lys particles are carried and deposited towards the center.

As we have mentioned already, the mound structure is formed by carrying and depositing the free Lys particles during the fluid front movement. As such, the increase of *ϕ* also triggers the upsurge of the overall number of particles, resulting in the piling of these particles. The formation of a dimple (depression) is probably when the Lys particles fall out of the solution. The presence of this dimple is not reported in any of the earlier works,^15,20^ probably due to the use of a relatively low concentration range. One can anticipate the dimple or the mound as the optical illusion, nonetheless, this is not the case. We did the height profile-like imaging with Sensofar microscopy to confirm the mound and dimple structures; however, we could not calibrate sufficiently to get the exact height measurements. It is also observed that the mound structure is situated almost at the center. This is due to the fact that the circular droplet shape prefers the symmetry for the fluid front movement. To confirm this fact, we pipetted the solutions in a strip-like geometry on the glass substrate. We followed the fluid front movement from both ends of the strips; however, we did not observe any mound or dimple structure. Furthermore, we assumed that this structure is likely to be shifted to some extent (not forming perfectly at the center of the droplet) due to the droplet’s circularity. However, no general trend is observed while measuring it in the asymmetrical (or oval) droplets.

#### 3.2.2 Quantification of the cracks

**Fig. 6:**
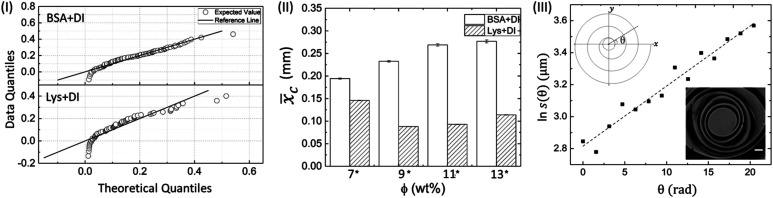
(I) Q–Q plot of BSA and Lys droplet at *ϕ* of 7 wt% displaying the skewed data points. (II) The histogram depicting the comparison of the mean crack spacing (*x̄*_c_) at different *ϕ* among the proteins in the peripheral regions. Significant pairs (BSA and Lys) are marked with an asterisk [*] at each *ϕ*. The error bars correspond to the standard error. (III) A characteristic linear fit of a spiral crack trajectory at *ϕ* of 13 wt% in Lys droplet. The upper inset shows the schematic diagram of the spiral cracks projected on the *xy* plane, in which *s*(*θ*) is the radial distance from the spiral center and *θ* is the angle starting from zero in an anticlockwise direction. The lower inset shows an example of a spiral focused at 50× objective lens, with a scale bar of length 10 μm.

Fig. 6(I) displays the Q–Q plot at *ϕ* of 7 wt% and confirms the non-normal distribution of the mean crack spacing (*x*_c_). The representative plots also suggest that the cracks are not equally distributed in different protein droplets. The figures indicate that the outliers have surfaced in the form of skewed data points. The outliers (depicted by three circles deviating from the reference line in the Q–Q plot) are not ideal considerations from a statistical perspective since these violate one of the assumptions for the *t*-test (parametric); nonetheless, in our case, there is no good reason to consider these outliers as invalid samples. To counter the non-normal distributions of the mean crack spacing, a non-parametric Mann–Whitney *U* test (an alternative to the parametric *t*-test) was preferred to examine the (significant) differences in terms of *x*_c_ values among the different protein droplets at different *ϕ*. In this study, the mean rank test is chosen over the median (for the visual inspection) because (a) the number of cracks are observed to be different in both protein droplets; and, (b) the number of samples is relatively large.

In the Mann–Whitney *U* test, the protein was kept as the categorized factor (independent factor, with two levels, BSA and Lys) and *x*_c_ as a dependent variable at different *ϕ*. All the histograms are expressed as the mean ± standard error (SE). The differences where *p* ≤ 0.05 are considered to be significant in this study. A detailed report of the statistical test (*U*, *z*, and *p* values) is shown in Table T5 of the ESI.†Fig. 6(II) shows *x̄*_c_ for the BSA and Lys droplets at each *ϕ*. The results confirm our morphological observations that the crack patterns in the BSA and Lys droplets are different from each other, resulting in a significant difference in *x̄*_c_ in the peripheral regions at every *ϕ* (the presence of crack spacing at every *ϕ* is significantly higher in Lys). The visual observations could be considered an effective way to determine the differences in terms of *x̄*_c_; however, the statistical test helps us to distinguish the data fluctuations borne out due to the experimental conditions. It is observed that *x̄*_c_ in the Lys droplets varies from 0.08 to 0.15 mm without any trend when the *ϕ* is increased. In contrast, *x̄*_c_ in the BSA droplet varies from 0.19 to 0.27 mm, and suggests a proportionate distribution; *i.e.*, *x*_c_ increase with increasing *ϕ*. The release of the available stress is mostly unidirectional in the BSA droplets, resulting in a uniform crack pattern in the peripheral ring. The uniform crack spacing resulted in an increased *x*_c_ when *ϕ* is also increased. However, in Lys droplets, the stress is relieved from all directions resulting in the distribution of small to large cracks spread throughout the droplet, affecting *x̄*_c_ at each *ϕ*.

Fig. 6(III) shows a characteristic linear fit of a spiral crack trajectory at *ϕ* of 13 wt% in the Lys droplet. The domains containing the spiral cracks in the Lys droplets are 3D and it is not possible to observe all the spiral lines simultaneously with the droplet surface. The lack of information about the *z* plane prompted us to represent these spirals on the *xy* plane (2D). The expression of the logarithmic spiral is in polar coordinates: *s*(*θ*) = *a*e^*bθ*^. The assumption of this logarithm leads to ln *s*(*θ*) = ln *a* + *bθ* where, *s*(*θ*) is the distance from the spiral center, and *θ* is the angle which is in an anti-clockwise direction from the *x* axis, not restricted to 2π. The final theta corresponds to the angle made by the *x* axis and the outermost spiral line. The schematic diagram of a 2D spiral is shown in the upper inset. The logarithmic parameters are “*a*”, which is the apparent length of the spiral, and “*b*”, which controls the tightness and predicts the direction of the spiral. A lower value of “*b*” means the spiral has more revolutions and hence, more tightness in the spiral shape. No preference of clockwise or counter-clockwise direction in the spirals is observed in any of these droplets. This prompted us to generalize the direction by flipping all the required images so that the spirals would be consistent every time with the starting spiral revolution line lying at zero degrees as shown in the lower inset. The linear fit between ln *s*(*θ*) and *θ* with *R*^2^ of 0.957 confirms that the spirals in the Lys droplets are in the form of logarithmic spirals. An oscillation of the data points is obtained due to the presence of the irregular, polygonal-shaped domains. The overall shape of the spirals for different *ϕ* is almost the same; however, the trajectories are influenced by the material and fracture properties (a similar phenomenon is observed in ref. 29 and 30). The spirals at *ϕ* of 13 wt% are shown in Fig. S1 of the ESI,† and various parameters of the spiral crack analysis at *ϕ* of 11 and 13 wt% are reported in Table T6 of the ESI.† Spirals with very few revolutions in *ϕ* of 13 wt% are also observed. The value of “*b*” is found to be in the range of 0.0376 to 0.0548 μm rad^−1^ – a narrow range implying that the tightness is probably insensitive to the concentration of proteins; however, a detailed trend of “*b*” requires the examination of more levels *ϕ*.

#### 3.2.3 A mechanical interpretation

It is reported in the earlier subsection that each droplet is pinned to the substrate throughout the drying process. The particles in the droplet are adsorbed on the substrate, and simultaneously are carried towards the periphery. With further water-loss from the droplet, the protein particles are deposited in such a way that it creates a film. These particles are accumulated in the layers and might be influenced by a shear-mode or mode II (the stress is applied parallel to the plane). However, this influence is almost negligible as the top surface of the film still contains enough water to evaporate. This water evaporates during the fluid front movement, and the tensile stressed fields are generated when the droplet is almost devoid of water.

**Fig. 7:**
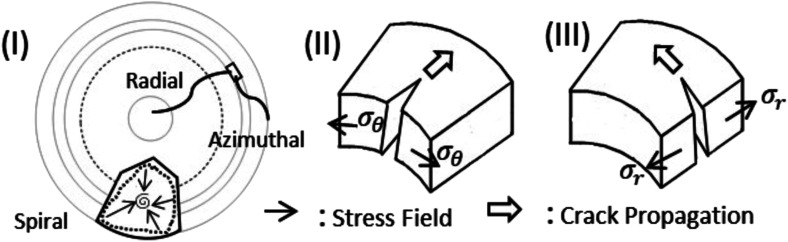
Schematic illustration explaining the nature and propagation of cracks in both protein droplets. (I) Top-view of a droplet shows radial and azimuthal cracks. A spiral crack is also shown in a polygonal crack domain. An element is focused on displaying crack propagation to get a notion of stress. Mode I (tensile mode) in which (II) stress (*σ*_θ_) acts normal to the radial crack and (III) stress (*σ*_r_) acts normal to the azimuthal crack.

Fig. 7 indicates that two types of tensile stress (mode I) are involved in propagating the radial and azimuthal cracks in the protein droplets. *σ*_θ_ and *σ*_r_ are the stresses that act normal to the radial crack and azimuthal crack, respectively. In both the droplets, a directional growth, *i.e.*, a radial crack, was initially observed to propagate from the periphery of the droplet (for example, see Fig. 2(I)c). Therefore, it indicates that the stress acts along the fluid front, normal to the radial crack, *i.e.*, *σ*_θ_ is dominant initially. It is known from Griffith’s hypothesis that the moment when the available stress in the film exceeds the critical stress, the excess stress is released by virtue of the crack propagation.^31,32^ This film height could be one of the reasons for the cracks appearing in the peripheral ring first, and then proceeding towards the central region in every protein droplet in general. We attempted height profile-imaging with Sensofar microscopy; however, we could not calibrate sufficiently to get the exact height measurements. The crack propagation could also be characterized based on the opening of the cracks and the distance of the crack tip as a function of *ϕ*. However, this is not possible with the current set-up as the time-lapse images are captured with an 8 bit camera and can only be taken every 2 s.

After the formation of the radial cracks, a few are curved azimuthally and the remaining ones join the neighboring cracks. However, it is observed that the stressed fields are dependent on the nature of protein particles. The crack propagation is stopped in BSA droplets, and a hair-like crack is developed without invading the central region of the droplet (Fig. 4d–g). The cracks form almost no connected domains. Our assumption is that the propagation of the cracks is stopped when the film thickness is less than the critical crack thickness resulting in zero cracks in the central region of every BSA droplet.

Lys, on the other hand, is a loosely-compacted and a low molecular weighted protein. The cracks are spread all over the droplet at all *ϕ* except 1 wt%. The presence of mound and dimple structures in the central region creates a gradient in the film thickness (highest at the periphery, lower at the center, and lowest at the middle region) during the drying process. This thickness gradient is enough to meet the crack propagation criteria and joins the crack lines from the center to the periphery. The stress fields act from all directions (*σ*_r_ and *σ*_θ_) leading to a chaotic system. No cracks are observed in the central region at 1 wt% (Fig. 4h) due to the presence of a few Lys particles in the middle region, which reduces the film thickness from the critical crack thickness. There are mostly well-connected polygonal domains in the Lys droplets at *ϕ* of 3 wt% and above (Fig. 4i–n). The cracking leads to a subsequent process of delamination at *ϕ* of 5 wt% and 7 wt% near the periphery of the droplet (Fig. 4j and k). Adhesion energy persists between the protein particles and the glass (substrate). As soon as the stored elastic energy in the domain overcomes this adhesion energy, each domain buckled – curving outwards like a bowl (a similar process is observed in other studies as well^33–35^). The interference fringes of each fragmented domain imply that there is an air gap between the detached film and the substrate that forms a non-uniform adhering region in each domain present in the Lys droplet. This phenomenon is observed in highly-magnified images of the Lys droplets presented in Fig. 4j–n.

Thus, a spiral path, is initiated in the well-connected polygonal domain of the Lys droplets at *ϕ* from 9 wt% onwards (Fig. 4l–n). The spirals propagate to release the elastic energy stored in the fragmented domain. This phenomenon is observed from *ϕ* of 9 wt% in the Lys droplet, implying that there is not enough stored elastic energy in the fragmented domains below *ϕ* of 9 wt%. The formation of the spirals on the irregular (polygonal) domains in the Lys droplets has no radial cracks intersecting the spiral cracks, *i.e.*, no splitting of the spiral cracks is observed in the Lys droplet (Fig. 4m and n). This indicates that the size domains become so small that there is no available energy for the radial cracks to propagate. At *ϕ* of 9 and 11 wt% in the Lys droplets, it is observed that the corners of the domains act as the precursor (Fig. 4l and m). This means that a high-stress area is achieved; however, due to lack of sufficient energy, the spirals fail to form the shape of those observed at *ϕ* of 13 wt% (Fig. 4n). Most of the spirals are present on the peripheral ring of the droplet at *ϕ* of 11 and 13 wt% (Fig. 4m and n). This implies that the film height could be one of the necessary criteria to have spiral cracks only at specific *ϕ* in the Lys droplet.

Although the increase of the protein concentration directly increases the film thickness, the heavy weighted protein (BSA of ∼66.5 kDa) contains a lower number of particles forming a thinner film height than that observed in Lys (∼14.3 kDa) at the same initial concentration. We argue that the absence of well-connected domains makes it hard to buckle the protein film. As a result, the stored elastic energy can not be applied in all directions on the delamination front to propagate in the BSA droplet. This is evident when we studied these proteins at *ϕ* of 9 wt% in the presence of 5CB liquid crystal (LC). None of the LC was present in the crack lines, and all were carried underneath the domains in Lys. The center of each domain adhered to the substrate and therefore appeared black under crossed polarizing configuration.^22^ In contrast, some of the LC was found in the crack lines and was mostly distributed on the top of the BSA film.^23^ These studies indicate that the domains of the BSA adhered to the substrate, but, Lys did not do so.

Interestingly, a hierarchy is only observed in the Lys protein droplets. At the lowest *ϕ* (1 wt%), the cracks are present in the peripheral ring of the Lys droplets. The cracks are observed throughout the film from 3 to 13 wt%; however, there is no delamination process involved until *ϕ* of 3 wt%. At *ϕ* of 5 and 7 wt%, the delaminated cracks are observed, particularly in the ring. As *ϕ* increased from 9 to 13 wt%, the circular and spiral cracks appeared in the ring in addition to the delamination (Fig. 4h–n). Observation of these spirals might be a common phenomenon in polymeric systems;^29,36,37^ however, such phenomenon observed in Lys droplets is yet to be reported. This proves a similar unstructured, amorphous reminiscent behavior of the Lys protein.

This mechanical interpretation, thus, reveals the differences in the type of crack patterns observed in the BSA and Lys droplets and throws light on the reasons behind the existence of spirals in the Lys droplets at specific *ϕ*. Further, this mechanical interpretation can also be used to explain the crack patterns in the dried droplets of any bio-molecules.

## 4 Conclusions

This work showcases the self-assembly of proteins and demonstrates that the process of self-assembly is driven by the drying process. The findings of the experiments confirm that the nature of a protein plays an important role in deciding the drying evolution and the subsequent morphology. The consistent reduction of the contact angle during the drying process helps in identifying the different modes of evaporation. The relatively higher initial protein concentration used in this study facilitated the identification of a “dimple” on the mound-structure in the dried Lys droplets. This study further establishes the presence of a spiral crack pattern at the specific initial protein concentration in Lys droplets, which has not been reported in the literature so far. The non-parametric statistical tests facilitated the crack spacing quantification and confirmed the visual observations. This procedure of quantification may be used in broad disciplines to quantify different parameters and their effects.

It is to be noted that all the experiments in this paper have been performed in DI water, and the presence of ions in body fluids will influence the patterns to a great extent. However, this study can set a baseline for understanding multi-component systems such as proteins with the addition of various salts (ions), whole human blood, plasma serum, *etc.* when dried under uniform conditions (surface, humidity, temperature, droplet diameter, *etc.*). The resulting pattern of the drying droplets is expected to be a signature of the initial state, as observed in our study. Thus, the findings of this paper ensure that such information may potentially to be used for diagnostic screening in the near future.

## Conflicts of interest

There are no conflicts to declare.

## Supplementary Material

RA-010-D0RA01748E-s001

RA-010-D0RA01748E-s002

RA-010-D0RA01748E-s003
