# Supplementary material for: A comparative study of the drying evolution and dried morphology of two globular proteins in de-ionized water solutions
Source: RSC Adv. 2020 Apr 30;10(29):16906–16. doi: 10.1039/d0ra01748e (PMC9053175; doi:10.1039/d0ra01748e)
Supplement: RA-010-D0RA01748E-s001 [file RA-010-D0RA01748E-s001.pdf]

Electronic Supplementary Material (ESI) for RSC Advances.
This journal is © The Royal Society of Chemistry 2020

SUPPLEMENTARY SECTION

I. DRYING EVOLUTION

The measured and the fitting parameters of drying protein droplets (BSA+DI and Lys+DI) are tabulated.

TABLE T1. Data from $\theta(t)$ vs. t graph of drying droplet at each ϕ (initial protein concentration in wt%) for BSA+DI with fitting parameters: θ_0 (contact angle at $t = 0$ in degrees), $1/\tau$ (characteristic rate in s^{-1}), and R^2 (adjusted R-square of the fit).

ϕ (wt%)	θ_0 (°)	$1/\tau \times 10^{-3}$ (s^{-1})	R^2
1	31.43 ± 0.04	1.560 ± 0.004	0.998
5	39.96 ± 0.08	1.500 ± 0.005	0.995
9	39.10 ± 0.07	1.510 ± 0.005	0.996
13	42.90 ± 0.07	1.540 ± 0.004	0.997

TABLE T2. Data from $\overline{r}(t)$ vs. t graph of drying droplet at each ϕ (initial protein concentration in wt%) for BSA+DI with measured parameters: R (radius of droplet in mm), w (rim width in mm), t_d (time in seconds after which the contact line radius begins to shrink from droplet radius), t_s (time in seconds at which two linear fits merge); and fitting parameters: m_1 (slope of first linear fit in μm/s), R_1^2 (adjusted R-square of first linear fit), m_2 (slope of second linear fit in μm/s), R_2^2 (adjusted R-square of the second linear fit). The negative sign in the slope values corresponds to the decrease in the radius of the contact line.

ϕ (wt%)	R (mm)	w (mm)	$t_d \pm 30$ (s)	$t_s \pm 20$ (s)	m_1 (μm/s)	R_1^2	m_2 (μm/s)	R_2^2
1	1.265 ± 0.025	0.3194 ± 0.043	254	n/a	-1.2	0.9604	n/a	n/a
3	1.129 ± 0.025	0.292 ± 0.072	307	n/a	-0.9	0.980	n/a	n/a
5	1.215 ± 0.028	0.507 ± 0.051	186	470	-0.8	0.979	-2.9	0.978
7	1.135 ± 0.036	0.465 ± 0.071	312	524	-0.7	0.983	-2.8	0.967
9	0.814 ± 0.017	0.378 ± 0.029	224	429	-1.2	0.977	-3.4	0.993
11	1.08 ± 0.016	0.543 ± 0.022	225	503	-1.0	0.996	-2.6	0.967
13	0.854 ± 0.014	0.462 ± 0.021	235	477	-1.1	0.983	-2.4	0.970

TABLE T3. Data from $\theta(t)$ vs. t graph of drying droplet at each ϕ (initial protein concentration in wt%) for Lys+DI with fitting parameters: θ_0 (contact angle at $t = 0$ in degrees), $1/\tau$ (characteristic rate in s^{-1}), and R^2 (adjusted R-square of the fit).

ϕ (wt%)	θ_0 ($^\circ$)	$1/\tau \times 10^{-3}$ (s^{-1})	R^2
1	43.83 ± 0.15	1.500 ± 0.008	0.987
5	36.64 ± 0.01	0.900 ± 0.001	0.999
9	41.89 ± 0.10	1.400 ± 0.005	0.994
13	40.38 ± 0.08	1.140 ± 0.004	0.994

TABLE T4. Data from $\overline{r}(t)$ vs. t graph of drying droplet at each ϕ (initial protein concentration in wt%) for Lys+DI with measured parameters: R (radius of droplet in mm), w (rim width in mm), t_d (time in seconds after which the contact line radius begins to shrink from droplet radius), t_s (time in seconds at which two linear fits merge); and fitting parameters: m_1 (slope of first linear fit in μm/s), R_1^2 (adjusted R-square of first linear fit), m_2 (slope of second linear fit in μm/s), R_2^2 (adjusted R-square of second linear fit). The negative sign in the slope values corresponds to the decrease in the radius of the contact line.

ϕ (wt%)	R (mm)	w (mm)	$t_d \pm 30$ (s)	$t_s \pm 20$ (s)	m_1 (μm/s)	R_1^2	m_2 (μm/s)	R_2^2
1	0.803 ± 0.017	0.195 ± 0.037	395	459	-1.0	0.987	-17.2	0.974
3	0.895 ± 0.014	0.320 ± 0.043	492	655	-1.0	0.973	-14.2	0.971
5	0.919 ± 0.020	0.383 ± 0.033	320	569	-1.0	0.974	-11.7	0.987
7	1.213 ± 0.028	0.411 ± 0.073	427	642	-0.9	0.978	-12.0	0.999
9	1.149 ± 0.034	0.491 ± 0.048	199	490	-0.9	0.941	-11.8	0.989
11	1.095 ± 0.016	0.481 ± 0.075	213	526	-1.1	0.955	-11.3	0.964
13	1.153 ± 0.016	0.438 ± 0.061	303	639	-1.1	0.948	-8.4	0.985

II. STATISTICAL ANALYSIS

In this section, the outcome of Mann-Whitney U test with the parameters U, z, p are tabulated. An asterisk [*] indicates that the interaction between the respective pairs (between BSA and Lys droplets) are significantly different at different initial protein concentration ϕ. The difference will be said statistically significant in terms of x_c, if p (calculated prob-

ability) is less than α (level of significance) being 0.05. This tells that the crack spacing is significantly different for BSA and Lys at every concentration ϕ.

TABLE T5. Outcome of Mann-Whitney U test with an asterisk [*] indicating significant interaction between Lys, and BSA in terms of x_c at different ϕ (wt %).

ϕ (wt%)	U	z	p
7	2893.500	-2.119	0.034*
9	2666.500	-5.331	$\leq 0.001^*$
11	1732.500	-5.752	$\leq 0.001^*$
13	1643.500	-5.439	$\leq 0.001^*$

III. SPIRAL CRACKS

In this section, the spirals which are found to be prominently present in the layer of lysozyme droplets at ϕ of 13 wt% are shown. The fitting parameters are tabulated for each spiral. The different spirals are focused by using 50× objective lens. There is no preference of clockwise or counter-clockwise found in each droplet, hence, we have generalized the direction of the spiral by flipping the spiral images so that the spirals become consistent every time with the starting spiral revolution line.

TABLE T6. Characteristic fitting parameters of spiral cracks at ϕ of 11, and 13 wt% for $\ln s(\theta) = \ln a + b\theta$ are tabulated: "b" denotes the spiral tightness in μm/rad, and "a" denotes apparent length in μm. $s(\theta)$ is the distance from the spiral center, and θ is the angle which is not restricted to 2π.

ϕ (wt%)	Spiral no	Spiral parameters b (μm/rad)	a (μm)	R^2
11	1	0.0548	13.1800	0.9460
13	1	0.0398	12.3407	0.9002
13	2	0.0437	13.3631	0.9373
13	3	0.0359	14.8322	0.9521
13	4	0.0376	16.7049	0.9569

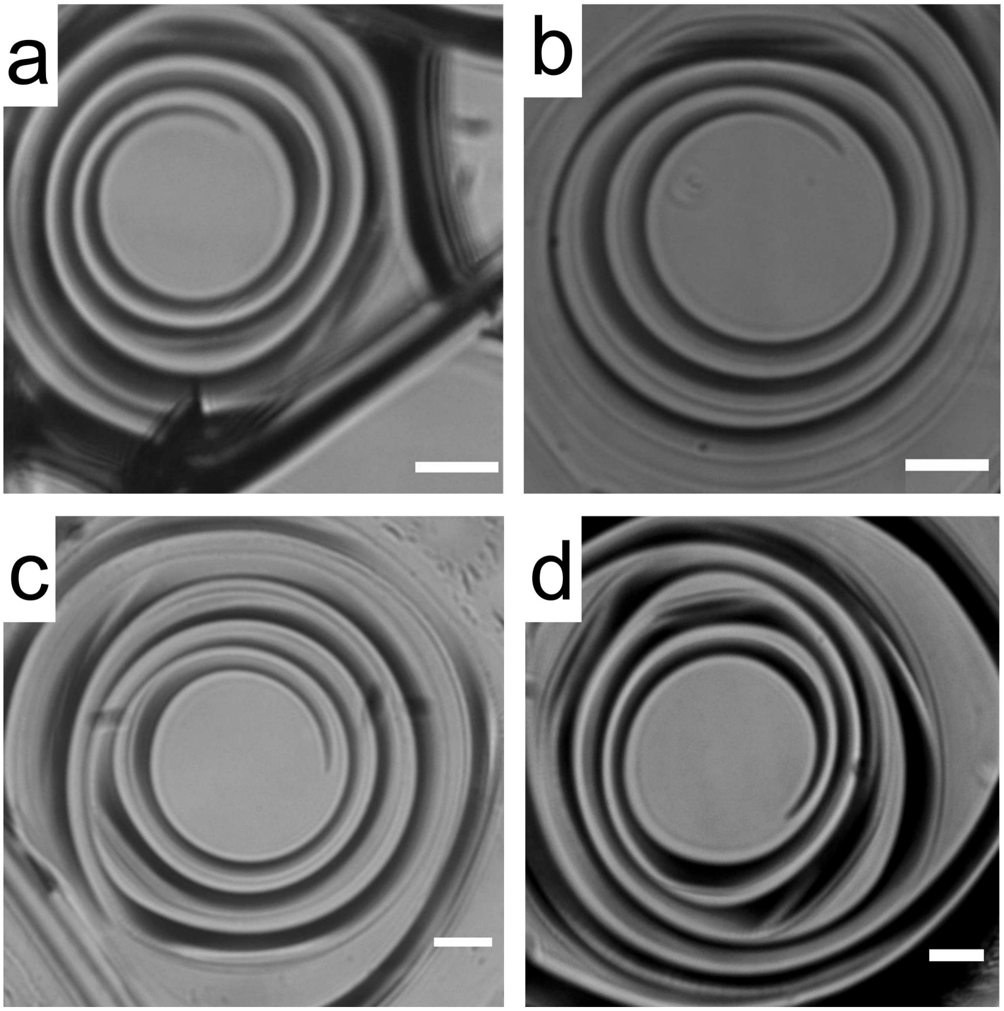

FIG. S1. (a)-(d) show the spirals from 1 to 4 are shown at ϕ of 13 wt% respectively, with the scale bar of length 10 μm.
